# On *Stenocypris* (Crustacea, Ostracoda) species from Yunnan Province, southwestern China, with a description of a new species

**DOI:** 10.3897/zookeys.1284.195403

**Published:** 2026-07-10

**Authors:** Dayou Zhai, Robin James Smith, Huijuan Mai, Ping Jiang, Kewei Zeng

**Affiliations:** 1 Yunnan Key Laboratory for Palaeobiology, Institute of Palaeontology, Yunnan University, Kunming 650500, China MEC International Joint Laboratory for Palaeobiology and Palaeoenvironment, Yunnan University Kunming China; 2 MEC International Joint Laboratory for Palaeobiology and Palaeoenvironment, Yunnan University, Kunming 650500, China Yunnan Key Laboratory for Palaeobiology, Institute of Palaeontology, Yunnan University Kunming China; 3 Lake Biwa Museum, 1091 Oroshimo, Kusatsu, Shiga 525-0001, Japan Lake Biwa Museum Kusatsu Japan

**Keywords:** Aquatic habitat, biogeography, natatory setae, oriental zoogeographical region, river basins, Yunnan–Guizhou Plateau

## Abstract

Surveys of waterbodies in Yunnan Province have yielded six species of the non-marine ostracod genus *Stenocypris* Sars, 1889, two of which have been previously described (*Stenocypris hirutai* Smith & Kamiya, 2006 and *S. orientalis* Victor & Fernando, 1981). One species is described as new, *Stenocypris menghaiensis***sp. nov**., and the other three are left in open nomenclature due to the scarcity of specimens. The newly described species is similar to *S. orientalis* in valve shape and morphology of most appendages, but it has reduced natatory setae on the antennae, in contrast to the long natatory setae in *S. orientalis*. Reduced antennal natatory setae are a relatively uncommon feature of the genus, found in only five of the 40 previously described species, but of the six *Stenocypris* species recorded in this study, a total of four have reduced natatory setae. This is probably a result of the habitats targeted, as reduced natatory setae are often associated with lotic habitats, such as spring discharges and riverine environments, but it could also indicate that the genus has a relatively high colonization rate of lotic environments in southwestern China. We highlight several morphological characters that are potentially useful for the taxonomy of the genus, including the width of the posterior calcified inner lamella, the ventral groove on the left valve, carapace surface micro-ornamentation, length of antennal natatory setae, and length ratios of setae on the sixth limb.

## Introduction

Yunnan Province, belonging to the Yunnan–Guizhou Plateau in southwestern China, stretches from the Qinghai–Tibetan Plateau in the north to the borders of Myanmar, Laos, and Vietnam in the south, covering an elevation range of ca 100–6750 m. Previously, more than 30 named extant ostracod species have been reported from this province, while others remain in open nomenclature or have only been reported as disarticulated valves from lake sediments (e.g., Yu 2014; Yu et al. 2022; Zhai et al. 2023, 2024, 2025; Jiang et al. 2025; Wang et al. 2025). Most of these extant species are reported from the central and eastern parts of Yunnan, but ongoing surveys are expanding the coverage in the province. Amongst the fauna recovered during surveys in central, southern, and western Yunnan are six species of *Stenocypris*, which are the focus of this study.

The non-marine ostracod genus *Stenocypris* includes 40 living subjective species, found in all major zoogeographical regions except Antarctica (Meisch et al. 2024). Most species are large (1.2–2 mm), elongate and laterally compressed, and easily recognizable as *Stenocypris* by the conspicuous, numerous septa (small support structures) around the free margins of the valves. However, species identification within the genus is taxonomically challenging due to many brief original descriptions with incomplete illustrations of the appendages (e.g., Vávra 1895, 1897; Klie 1932), a generally conservative appendage morphology, the lack of males for most species (e.g., Smith et al. 2006; Ma and Yu 2018, 2020), and the tendency for parthenogenetic species to express relatively large amounts of carapace variation (Martens 2001). The taxonomy of *Stenocypris* has often focused on the lateral outline of the carapace, the lengths of marginal septa, and the morphology of the uropods, but these characters alone can be insufficient to discriminate species. To overcome these taxonomic hindrances, full, detailed descriptions are required, not only of all appendages, but also fine details of the carapace, some of which, such as micro-ornamentation, are best observed with scanning electron microscopy (e.g., Moonchaisook and Savatenalinton 2020).

This contribution aims to describe a new species and provide new records and notes on other *Stenocypris* species, some of which are left in open nomenclature due to the scarcity of specimens.

## Material and methods

### Sites and sampling

**Figure 1.**
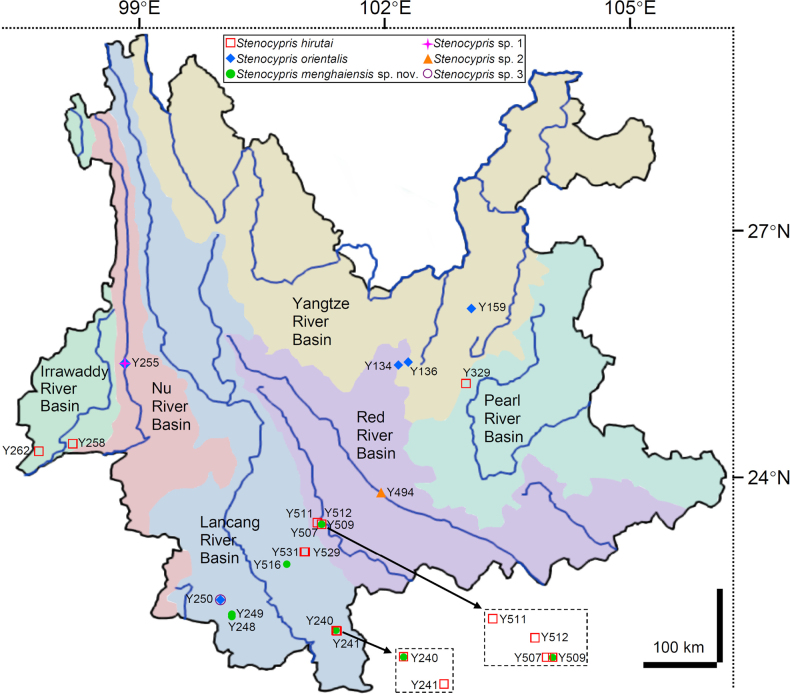
Map of Yunnan province of southwestern China, showing the distribution of sampling sites in different drainage basins. Redrawn from https://yunnan.tianditu.gov.cn/index.

During August 2017 and April 2025, ostracod specimens were collected from 20 sites distributed across six major river basins in Yunnan, southwestern China (Fig. 1, Table 1). The sites include both lentic and lotic habitats across a latitudinal range of ca 21°57'N–25°53'N, a longitudinal range of ca 97°48'E–103°05'E, and an altitudinal range of ~ 400–2100 m a.s.l. (Fig. 1, Table 1). Apart from two desiccated sites (see below), all the other sites were inundated by freshwater, with electrical conductivity between 101 and 610 µS cm^-1^ and pH between ~ 6.9 and 9.0. The waters were relatively warm, with temperature between ~ 17 °C and 30 °C.

**Table 1.** Data and the *Stenocypris* species found at each of the sampling sites from Yunnan Province, China. Note that not all the specimens are adults; juveniles were identified based on morphological comparison with the dissected adults. Dates of the samples are in the format of year-month-day. For explanations of abbreviations see Terminology and abbreviations.

**SN, and date**	**Habitat**	**GPS coordinates**	**Altitude (m a.s.l.)**	**EC (µS cm^-1^**)	**pH**	**T (°C**)	**Substrate**	**Vegetation**	*Stenocypris* species and number of individuals*
Y134, 2017-08-05	Wet rice field	25°11'08"N, 102°11'27"E	1820	244	na	23.0	Mud	Rice crops	*S. orientalis* (4)
Y134, 2022-03-10	Desiccated rice field	25°11'08"N, 102°11'27"E	1820	na	na	na	Mud	Rice stubble and abundant short grasses	*S. orientalis* (7)
Y136, 2017-08-05	Wet rice field	25°13'57"N, 102°18'09"E	1920	101	na	24.2	Mud	Rice crops	*S. orientalis* (1)
Y159, 2022-09-20	Wet barren field	25°52'43"N, 103°04'32"E	2080	449	7.73	23.8	Mud	Abundant macrophytes, dense grasses, and abundant filamentous algae	*S. orientalis* (6)
Y240, 2023-04-19	Ditch	21°57'24"N, 101°25'38"E	770	189	7.16	26.6	Mud and abundant plant remains	None	*S. hirutai* (2) and *Stenocypris menghaiensis* sp. nov. (10)
Y241, 2023-04-19	Ditch	21°57'24"N, 101°25'38"E	770	178	7.01	27.1	Abundant plant remains	Some grasses, extending from banks into water	*S. hirutai* (18)
Y248, 2023-04-20	Wet barren field crossed by stream	22°08'38"N, 100°09'14"E	1070	190	6.89	27.2	Mud	Dense grasses and abundant filamentous algae	*Stenocypris menghaiensis* sp. nov. (13)
Y249, 2023-04-20	Stream	22°08'38"N, 100°09'13"E	1070	202	7.76	27.0	Mud and gravel	A few filamentous algae	*Stenocypris menghaiensis* sp. nov. (7)
Y250, 2023-04-20	Desiccated rice field	22°19'52"N, 100°01'09"E	1160	na	na	na	Dry hard mud	Rice stubble and some short grasses	*S. orientalis* (15) and *Stenocypris* sp. 3 (1)
Y255, 2023-04-25	Stream	25°12'47"N, 098°50'46"E	730	396	8.08	29.7	Mud and fallen leaves	Dense grasses on edges of stream	*S. orientalis* (10) and *Stenocypris* sp. 1 (1)
Y258, 2023-04-26	River	24°13'34"N, 098°12'42"E	790	374	7.74	26.0	Sand	Abundant macrophytes and filamentous algae	*S. hirutai* (3)
Y262, 2023-04-26	Stream	24°08'43"N, 097°47'37"E	940	136	6.92	28.3	Mud	None	*S. hirutai* (12)
Y329, 2024-06-12	Pond	24°58'06"N, 103°00'38"E	1770	610	8.83	28.3	Mud and plant remains	Dense grasses on edge of pond	*S. hirutai* (2)
Y494, 2025-04-01	River	23°37'10"N, 101°58'28"E	410	597	8.66	19.8	Gravel and mud	Abundant filamentous algae in river, dense grasses along banks	*Stenocypris* sp. 2 (1)
Y507, 2025-04-02	Ditch	23°15'12"N, 101°14'22"E	860	534	9.02	20.9	Sand	Abundant filamentous algae in ditch, dense grasses along banks	*S. hirutai* (20)
Y509, 2025-04-02	River	23°15'11"N, 101°14'25"E	860	507	9.04	21.1	Mud	Dense grasses along one bank, some filamentous algae on riverbed	*S. hirutai* (30) and *Stenocypris menghaiensis* sp. nov. (1)
Y511, 2025-04-02	Stream	23°18'33"N, 101°13'17"E	1090	278	8.46	18.6	Gravel and mud	None	*S. hirutai* (7)
Y512, 2025-04-02	Stream	23°16'54"N, 101°14'06"E	870	399	8.38	20.8	Mud	Dense grasses along banks	*S. hirutai* (138)
Y516, 2025-04-03	Stream	22°45'22"N, 100°49'26"E	1290	210	7.83	17.5	Mud, sand, and fallen leaves	None	*Stenocypris menghaiensis* sp. nov. (2)
Y529, 2025-04-04	Stream	22°54'44"N, 101°02'14"E	1290	291	7.94	17.4	Mud, gravel, and fallen leaves	None	*S. hirutai* (37)
Y531, 2025-04-04	Stream	22°54'44"N, 101°02'08"E	1310	249	8.22	17.8	Mud and fallen leaves	A few grasses along banks	*S. hirutai* (1)

*: Both adults and juveniles were counted in this table, although only adults were described in the Systematic description section. All the sites are shallower than 1 m. The information on the environmental parameters and the ostracods of the site Y134 were previously published in Zhai et al. (2024), and the *Stenocypris* specimens were misidentified as *S. major*. See text for details.

One of the sites (Y134) was visited twice, yielding two samples, thus there are a total of 21 samples for the 20 sites (Table 1). One of the samples from Y134 and the sample from Y250 (both rice fields) were collected during the dry phase. These samples were cultured in the laboratory for ostracods (for details of the sample from Y134 see Zhai et al. 2024; the culturing procedure of the sample from Y250 was the same). All other samples were taken in wet habitats, where the ostracods were collected with a sieve with a 180-μm mesh size and were preserved in 70% ethanol.

### Laboratory analyses

In the laboratory, the ostracods were picked from the samples under a Jiangnan JSZ6S stereomicroscope, and dissected prior to morphological examination. On a glass slide, before a small drop of Hydro-Matrix® (Dr. Richard Rudnicki, Micro Tech Lab, Austria) was added, the valves were removed and temporarily placed in a tiny cup filled with tap water in order to avoid dissolution of the calcareous valves by the acidic Hydro-Matrix. The valves were brush-cleaned and stored dry in laser-cut micropaleontological slides (Dr. Marian Golej, Kreativika Design, Slovakia). All specimens are deposited in the Yunnan Key Laboratory for Palaeobiology, Institute of Palaeontology, Yunnan University, Kunming, China.

Both the soft parts and the valves were examined with a Shangguang XSP-12CA transmitted light microscope and were photographed with a Canon EOS 5D Mark IV camera connected to this microscope. Photographs of the valves were taken from both the exterior view and the oblique-dorsal view. In the former case, each valve was placed convex-upwards on a horizontal glass slide, offering observation of the outline, the width of CIL, and the general morphology of the septa. In the latter case, the valve was rested on its ventral margin, tilted slightly exterior-ward, offering an intero-dorsal aspect to the microscope, and allowing clear observation of the ventral marginal structures, but especially those in the antero-ventral and postero-ventral areas. Line drawings of the soft parts were produced in Microsoft PowerPoint 2019, based on both the microscopic photographs and the simultaneous examination of the chaetotaxy through the microscope. Selected appendicular structures (see Zhai et al. 2017) were measured with the ocular ruler of the XSP-12CA microscope (Suppl. material 1). The valve outlines were digitized from the XSP-12CA microscopic photographs using the software TPSDIG2 (v. 2.31) (Rohlf and Bookstein 1990; Rohlf 2017) (Suppl. material 2). Inter-specimen morphological dissimilarities were calculated based on the appendicular and valve outline data, following the procedures of Zhai et al. (2017) and Wang et al. (2022), respectively, resulting in dissimilarity matrices of the specimens (Suppl. material 3). In order to visualize the inter-specimen morphological difference, Principal Coordinate Analysis (PCoA) was performed on the forward-selected normalized parameters (Suppl. material 4, in which the dissimilarity values with the two most outlying specimens in each matrix in Suppl. material 3 were selected, in addition to other measurements) using the software R (v. 4.3.1, The R Core Team 2023). The R package vegan (v. 2.6-4, Oksanen et al. 2022) was utilized to assist the analysis. Some valves were also photographed with a Hitachi Regulus 8100 scanning electron microscope for illustration.

### Terminology and abbreviations

Abbreviations used in the text, figures, and tables are as follows. **A1**—antennule; **A2**—antenna; **as**—accompanying seta of the grouped setae; **ca**—cavity between septa; **CIL**—calcified inner lamella; **Cp**—carapace; **dor**.—dorsal view/side; **EC**—electrical conductivity; **exs**—exopodal setae; **ext**.—exterior view/side; **GL**—genital lobe; **gr**—groove; **gs**—grouped setae on mandibular palp; **H**—height; **ids**—interdental seta; **IL**—inner list; **IM**—inner margin (boundary between calcified and uncalcified inner lamellae); **int**.—interior view/side; **L**—length; **LV**—left valve; **L5/L6/L7**—fifth/sixth/seventh limb; **Md**—mandible; **Mx**—maxillula; **na**—not available; **N**—number of values available for calculation; **RO**—Rome organ; **RPC**—radial pore canal; **RV**—right valve; **SD**—standard deviation; **SEM**—scanning electron microscope; **sep**—septum/septa; **SN**—site number; **T**—temperature of water; **tlb**—tooth lobes; **UIL**—uncalcified inner lamella; **Ur**—uropod; **ven**.—ventral view/side; **W**—width; **WD**—water depth. Terminology of the appendage chaetotaxy follows Broodbakker and Danielopol (1982), Martens (1987) and Meisch (2000). Numbering of the segments of the A1 follows an eight-segmented model (e.g., Smith and Kamiya 2007), which was slightly modified from Smith and Tsukagoshi (2005). Designations of Gm and GM claws of the A2 follow Martens (1987: fig. 2), Meisch (2000: fig. 6), and Karanovic (2012: 26), with the more interior claw designated as GM while the more exterior one as Gm.

## Results

A total of six *Stenocypris* species were recovered in the present study (Table 1). The two most abundant species were *Stenocypris hirutai* Smith & Kamiya, 2006, found at 11 sites, and *Stenocypris orientalis* Victor & Fernando, 1981, found at five sites. A new species of *Stenocypris*, found at five sites, is described below. Additionally, three other species are kept in open nomenclature because only a single specimen of each was recovered.

### Systematic descriptions

**Order Podocopida Sars, 1866**

**Superfamily Cypridoidea Baird, 1845**

**Family Cyprididae Baird, 1845**

**Subfamily Herpetocypridinae Kaufmann, 1900**

**Tribe Stenocypridini Ferguson, 1964**

**Genus *Stenocypris* Sars, 1889**

#### Stenocypris menghaiensis sp. nov.

Taxon classificationAnimaliaPodocopidaCyprididae

89FD5162-DA64-5BE1-BE89-4CDE527BB3D6

https://zoobank.org/3E5EBF82-B520-461B-9615-1E5621E6CFD5

**Figure 2.**
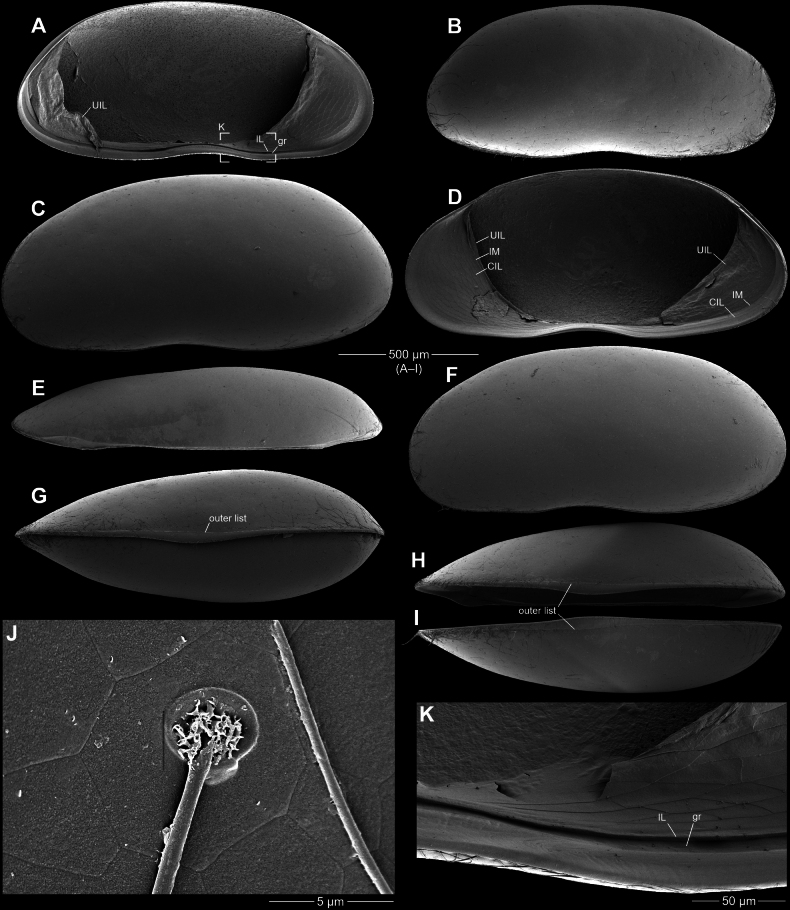
*Stenocypris menghaiensis* sp. nov., females. **A**. LV, int. (WOC696); **B**. RV, ext. (WOC696); **C**. LV, ext. (WOC477); **D**. RV, int. (WOC477); **E**. RV, dor. (WOC483, holotype); **F**. LV, ext. (WOC483, holotype); **G**. Cp, ven. (WOC722); **H**. LV, ven. (WOC714); **I**. RV, ven. (WOC714); **J**. Surface detail of (**B**), showing the polygonal pattern and a rim-bearing pore; **K**. Detail of marginal area of (**A**), showing the groove. Anterior to left for (**E, G–I**). See text for explanation of abbreviations.

**Figure 3.**
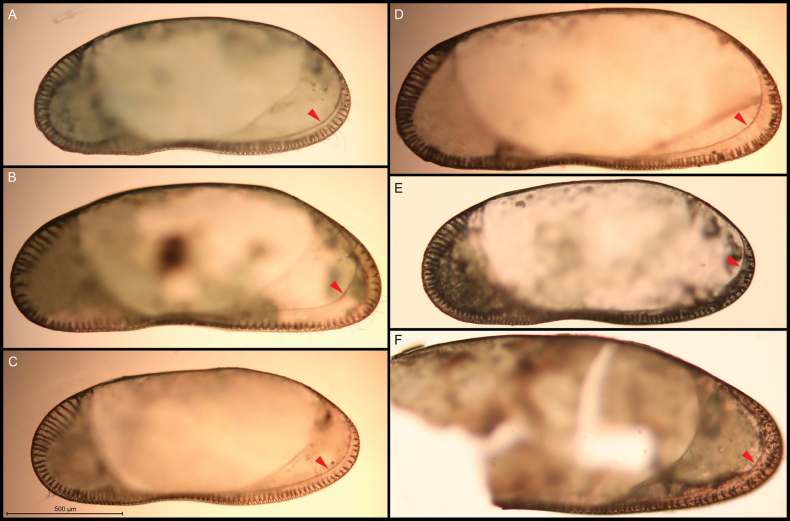
Exterior views of the female LVs of *Stenocypris* species. The general morphology of the septa and the width of the postero-ventral CIL are shown. Arrowheads point to the position of the IM. **A**. *Stenocypris menghaiensis* sp. nov. (WOC483, holotype); **B**. *S. hirutai* Smith & Kamiya, 2006 (WOC541); **C**. *S. orientalis* Victor & Fernando, 1981 (OSBK31); **D**. *Stenocypris* sp. 1 (WOC176); **E**. *Stenocypris* sp. 2 (WOC547); **F**. *Stenocypris* sp. 3 (WOC485, anterior part broken during preparation). Scale applies to all panels.

**Figure 4.**
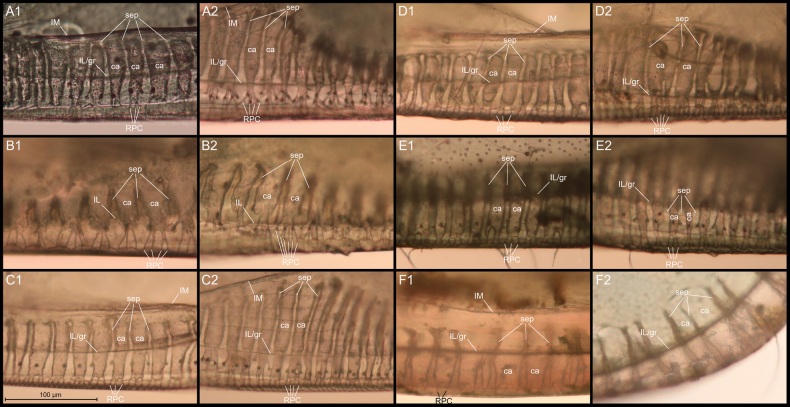
Detailed morphology of the marginal zone of the female LVs of *Stenocypris*. Each pair of photographs includes the postero-ventral (**A1, B1, ..., F1**) and the antero-ventral (**A2, B2, ..., E2**) areas, respectively. **F2** is the interior view of a fragment of the ventro-anterior part. All other panels are interior views of the ventral margin, which was accessed from the intero-dorsal view of the valve (Suppl. material 5; see Material and methods section for details). The specimens in **A1/A2–F1/F2** correspond to those in Fig. 3A–F, respectively. (**A1, A2**) *Stenocypris menghaiensis* sp. nov. (WOC483, holotype); (**B1, B2**) *S. hirutai* Smith & Kamiya, 2006 (WOC541); (**C1, C2**) *S. orientalis* Victor & Fernando, 1981 (OSBK31); (**D1, D2**) *Stenocypris* sp. 1 (WOC176); (**E1, E2**) *Stenocypris* sp. 2 (WOC547); (**F1, F2**) *Stenocypris* sp. 3 (WOC485). Scale applies to all panels.

Figs 2, 3A, 4A1–A2, 5, 6, Suppl. material 5: fig. 1A, Suppl. material 6

##### Type locality.

Site Y249 (Fig. 1, Table 1), stream in valley in Menghai County of Yunnan Province (22°08'38"N, 100°09'13"E, altitude 1070 m a.s.l.).

##### Type material.

***Holotype***: • dissected female (WOC483) from type locality. ***Paratypes***: • five dissected females (WOC477, WOC570, WOC602, WOC696, WOC714), from type locality and another four sites (Table 2).

##### Additional material.

Two undissected females (WOC722, WOC723) from site Y240 (Table 1). WOC722 with soft parts deteriorated to some extent.

##### Dimensions.

LV (*n* = 6) L 1287–1416 μm, H/L 0.439–0.450. RV (*n* = 4) L 1252–1385 μm, H/L 0.435–0.441. LV ~ 30–40 μm longer than RV of same individual (Table 2).

##### Diagnosis.

Small-sized *Stenocypris*. Septa well-developed, expanded at antero-dorsal and antero-ventral areas. CIL narrow at postero-ventral area, only slightly wider than septal band, without any inward expansion. Groove well-developed on LV, narrow ventrally, wider and shallower towards both ends. LV overlaps RV along free margins. Valve surface without striations, but with polygonal pattern observable under high magnification of SEM. A1 with pear-shaped RO. Swimming setae on A2 short, extending to approx. midway of penultimate segment. L6 with seta h3 more than twice as long as h1. Right Ur ramus with more pronounced spines than left Ur ramus. Size and number of spines on right Ur ramus variable among individuals.

##### Description of female.

***Cp*** (Figs 2, 3A) sub-reniform in lateral view. Dorsal margin gently curved, with highest part near or slightly behind mid-length. Anterior margin broadly rounded, with antero-dorsal section of RV slightly concave. Posterior margin narrowly rounded, with upper section nearly straight. Ventral margin concave. In interior view, LV with distinct ventral groove situated immediately below inner list. Groove narrow in ventral area, shallower and wider towards both ends (Fig. 2A, K). Anterior CIL wide, with polygonal patterns (Fig. 2A, D, K). Posterior CIL narrow (Figs 2A, 2D, 3A). Septa narrow proximally, becoming wider towards valve margin (Fig. 4A1–A2). Each cavity between septa branching into comparatively long radial pore canals. False pore canals present. Valve surface covered with polygonal pattern (Fig. 2J). Setal pores with rims. In dorsal (Fig. 2E) and ventral (Fig. 2G–I, Suppl. material 6: fig. 2B) views, greatest width situated at posterior ~ 1/3 to 1/4. In ventral view, septal band nearly symmetrical between two valves (Suppl. material 6: fig. 2B). LV overlaps RV on anterior, ventral, and posterior margins. Both valves with outer list (Fig. 2H, I), yet that on RV concealed by LV upon valve closure (Fig. 2G). Shell pale white or light cyan (Fig. 3A; Suppl. material 6: fig. 2A). For measurements, see Table 2.

**Table 2.** The valve sizes of the dissected adult females of *Stenocypris* from Yunnan Province, China. Measurements were made from photographs taken with a Shangguang XSP-12CA transmitted light microscope unless otherwise noted.

**Collection number**	**Taxon**	**Site**	**LVL (μm)**	**LVH (μm)**	**LVH/L**	**RVL (μm)**	**RVH (μm)**	**RVH/L**
WOC477	*Stenocypris menghaiensis* sp. nov.	Y248	1416	630	0.445	1385	607	0.438
WOC483^●^		Y249	1365	599	0.439	1328	578	0.435
WOC570		Y509	1374	612	0.445	na	na	na
WOC602		Y516	1412	636	0.450	na	na	na
WOC696		Y249	1287	567	0.441	1252	552	0.441
WOC714		Y240	1338	593	0.443	1308	571	0.437
WOC403	*Stenocypris hirutai* Smith & Kamiya, 2006	Y329	1641*	na	na	1610*	650*	0.404*
WOC499		Y258	1521	639	0.420	1491	613	0.411
WOC506		Y262	1633	653	0.400	1600	633	0.396
WOC537		Y240	1566	636	0.406	1517	605	0.399
WOC540		Y241	na	na	na	na	na	na
WOC541		Y241	1597	650	0.407	1565	620	0.396
WOC566		Y507	1593	657	0.412	1555	634	0.408
WOC568		Y507	1590	656	0.413	1543	630	0.408
WOC571		Y509	1688	700	0.415	1637	675	0.412
WOC572		Y509	1491	604	0.405	1458	586	0.402
WOC573		Y511	1784	728	0.408	1766	702	0.398
WOC574		Y511	1720	711	0.413	1682	684	0.407
WOC575		Y512	1871	753	0.402	1838	742	0.404
WOC633		Y529	1869	731	0.391	1844	728	0.395
WOC643		Y531	1820	732	0.402	1784	726	0.407
WOC706		Y262	1637	654	0.400	1593	634	0.398
WOC707		Y262	1509	623	0.413	1474	599	0.406
WOC713		Y529	1954	786	0.402	1923	780	0.406
WOC715		Y240	1564	645	0.412	1535	623	0.406
dyzoc788	*Stenocypris orientalis* Victor & Fernando, 1981	Y136	1442	634	0.440	1413	611	0.432
OSBK31		Y134	1415	599	0.423	1391	577	0.415
OSBK33		Y134	1499	620	0.414	1475	598	0.405
OSBK34		Y134	1389	593	0.427	1358	576	0.424
WOC122		Y159	1763	721	0.409	na	700	na
WOC146		Y134	1422	609	0.428	1394	586	0.420
WOC493		Y255	1439	595	0.413	1405*	585*	0.416*
WOC701		Y250	na	na	na	na	na	na
WOC705		Y255	na	na	na	na	na	na
WOC176	*Stenocypris* sp. 1	Y255	1662	706	0.425	1640	677	0.413
WOC547	*Stenocypris* sp. 2	Y494	1416^†^	628^†^	0.443^†^	1385^†^	596^†^	0.430^†^
WOC485	*Stenocypris* sp. 3	Y250	2055*	791*	0.385*	2018*	772*	0.383*

Some valves were damaged (WOC122, WOC403, WOC602), lost (WOC570), or were deformed due to poor calcification (WOC701, WOC705). Therefore, some dimensions are not available. ^●^: holotype. *: These were measured with a Jiangnan JSZ6S stereomicroscope. The valves were damaged before they could be examined with the XSP-12CA microscope. ^†^: Both valves of this specimen were measured from the images generated by a Hitachi Regulus 8100 scanning electron microscope. The RV was damaged before it could be examined with the XSP-12CA microscope.

**Figure 5.**
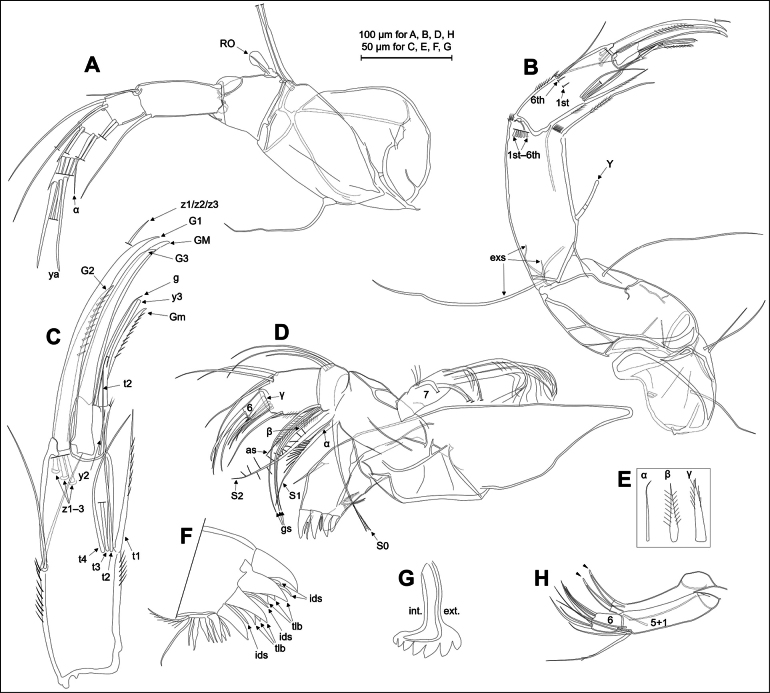
Soft parts of *Stenocypris menghaiensis* sp. nov., female. **A–C, H**. WOC483 (holotype); **D, E**. WOC477; **F**. WOC696; **G**. WOC602. **A**. A1; **B**. A2; **C**. distal part of (**B**); **D**. Md; **E**. Details of setae α, β and γ of (**D**); **F**. Details of masticatory processes of coxa; **G**. Food rake; **H**. Endopod and distalmost endite (setae other than tooth-bristles not shown) of Mx.

***A1*** (Fig. 5A) with eight segments. First two segments fused forming robust base, carrying one dorsal seta and two long ventro-apical setae. Third segment sub-trapezoidal, bearing one small dorsal seta. RO situated at sub-proximal position of this segment, relatively big, consisting of two sections (small basal section sometimes difficult to see), distally enlarged. Fourth segment elongate, with one dorso-apical seta extending to approx. end of terminal segment, and ventro-apical seta extending somewhat beyond fifth segment. Fifth and sixth segments sub-quadrate, each with two long dorso-apical setae and two long ventro-apical setae. Seventh segment slightly elongate, with four long setae on interior side and one short thin seta (α) on exterior side. Eighth segment slightly elongate, distally with three setae and one aesthetasc (ya). Ventral seta thick, distally slightly claw-like. Aesthetasc ya sub-equal to ventral seta in length. Two intermediate setae long.

***A2*** (Fig. 5B, C) first protopodal segment (coxa) short, bearing one postero-lateral seta and two ventro-apical setae. Second protopod segment (basis) robust, bearing one long ventro-apical seta. Exopod plate small, bearing three progressively shorter setae, longest one extending to approx. end of first endopodal segment. First endopodal segment elongate, with aesthetasc Y slender, situated behind mid-length. Swimming setae (setae 1–5 in Fig. 5B) sub-equal, extending to or slightly beyond mid-way of penultimate segment. Seta 6 sub-equal in length to swimming setae but slightly thicker. First endopodal segment also with one plumose ventro-apical seta extending to approx. end of terminal segment. Short stiff pseudochaetae present at dorso-apical and ventro-apical parts of this segment. Penultimate segment undivided, medio-ventrally with four t-setae, t2 and t3 being longer than t1 and t4 (Fig. 5C). Seta t1 plumose. Aesthetasc y1 not observed. Penultimate segment medio-dorsally with two unequally long setae. Setae z1–z3 extending to approx. tips of longest claw (G1). G2 slightly longer than 2/3 length of G1. G3 slightly shorter than G1. G1 and G3 smooth. G2 conspicuously serrated. Aesthetasc y2 situated ventro-apically on penultimate segment, slender, extending to approx. end of terminal segment. Terminal segment with GM, Gm, g, and branched y3. GM extending to tip of G1, smooth. Gm somewhat longer than 1/2 length of GM, serrated. y3 and g sub-equally long as Gm.

***Md*** (Fig. 5D–F) with coxa distally carrying four strong and ~ 3 slender masticatory processes (these slender processes difficult to observe in Fig. 5D because of curving of coxa). Interdental setae present. At least second and third masticatory processes with three lobes (Fig. 5F). Two slender setae present at end of masticatory process array. One smooth seta present on sub-apical part of anterior margin of coxa (Fig. 5D). Md palp (Fig. 5D) consisting of exopod-bearing basis and three endopodal segments. Exopod (vibratory plate) with seven unequal plumose rays. Basis ventro-apically with four setae: S0 distally setulose, S1 and S2 plumose, α slender, smooth (Fig. 5E). First endopodal segment dorsally with three unequally long setae, ventrally with three equally long, slender, smooth grouped setae (‘gs’ in Fig. 5D), shorter plumose accompanying seta (‘as’ in Fig. 5D), and short plumose seta β (Fig. 5D, E). Second endopodal segment dorsally with four setae, distally with four setae (including plumose γ, see also Fig. 5E), and ventrally with one long and one very short seta. Distal segment with three thick and three slender setae.

***Mx endopod*** (Fig. 5H) two-segmented. First segment elongate, bearing one lateral seta and five dorso-apical setae. Second segment sub-rectangular, bearing six setae. Tooth-bristles on distalmost gnathobasic endite smooth (arrowed in Fig. 5H). (Other setae on this endite and two proximal gnathobasic endites not illustrated.)

***Food rake*** (Fig. 5G) distally with ~ 7 processes, four exterior-most ones generally larger than other three.

**Figure 6.**
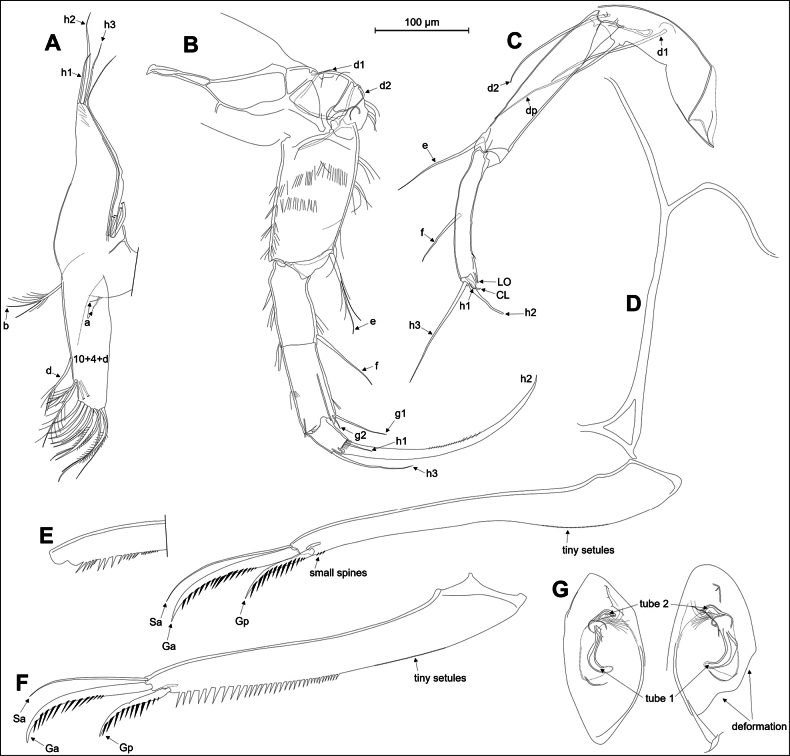
Soft parts of *Stenocypris menghaiensis* sp. nov., female. **A–D, F**. WOC483 (holotype); **E**. WOC570; **G**. WOC602. **A**. L5; **B**. L6; **C**. L7; **D**. Left Ur and attachment; **E**. Distal part of left Ur ramus, note the difference in the spine size between (**D**) and (**E**); **F**. Right Ur; **G**. GLs.

***L5*** (Fig. 6A) with two smooth setae a, and plumose setae b and d. Seta c absent. Gnathobasic endite with ten apical setae and four sub-apical setae situated between seta d and apical setae. Exopod with ~ 5 rays. Endopod with comparatively short setae h1–h3.

***L6*** (Fig. 6B) with five segments. Pseudochaetae present on every segment. First segment (protopod) with short seta d1 and somewhat longer seta d2. Second segment (first endopodal segment) robust, bearing plumose seta e. Third segment slender, bearing seta f that extends to ~ 3/4 of next segment. Fourth segment sub-equally long to third segment, bearing seta g1 and minute seta g2. Terminal segment sub-rectangular, only slightly trapezoidal, distally with row of stiff pseudochaetae and h1–h3. Seta h3 more than twice as long as seta h1. Claw h2 sub-equally long to total length of last three segments of L6, serrated (or bearing stiff setules) medially.

***L7*** (Fig. 6C) with three elongate segments. Basal segment (protopod) with sub-equally long setae d1 and d2, and longer dp. Second segment (first endopodal segment) with seta e extending to approx. midway of third segment. Third segment medially with short seta f and distally with pincer arrangement. Seta h1 very small. h2 nearly straight or slightly curved. Seta h3 sub-equally long to e, straight (not curved medially).

***Ur*** (Fig. 6D–F) asymmetrical. Right Ur ramus (Fig. 6F) with more pronounced spines than left Ur ramus (Fig. 6D, E). Number of spines on right Ur ramus between 22 and 33, averaging 28 (*n* = 6). Spines generally becoming larger towards distal end of ramus, size change being either gradual or somewhat discontinuous. Left Ur ramus with only setule-like spines in distal area (Fig. 6D), or with well-developed spines (Fig. 6E). Both Ur rami with tiny setules at sub-proximal area. In both Ur, Gp extending to midway of Ga, both strongly serrated. Sa slightly shorter than Ga. Sp absent. Ur attachment (Fig. 6D) with triangular loop distally.

***GL*** (Fig. 6G) sub-ovate and somewhat elongate, weakly sclerotized, not rarely deformed during dissection. Central area of GL with two sets of curved internal tubes.

Males unknown.

##### Differential diagnosis.

**Figure 7.**
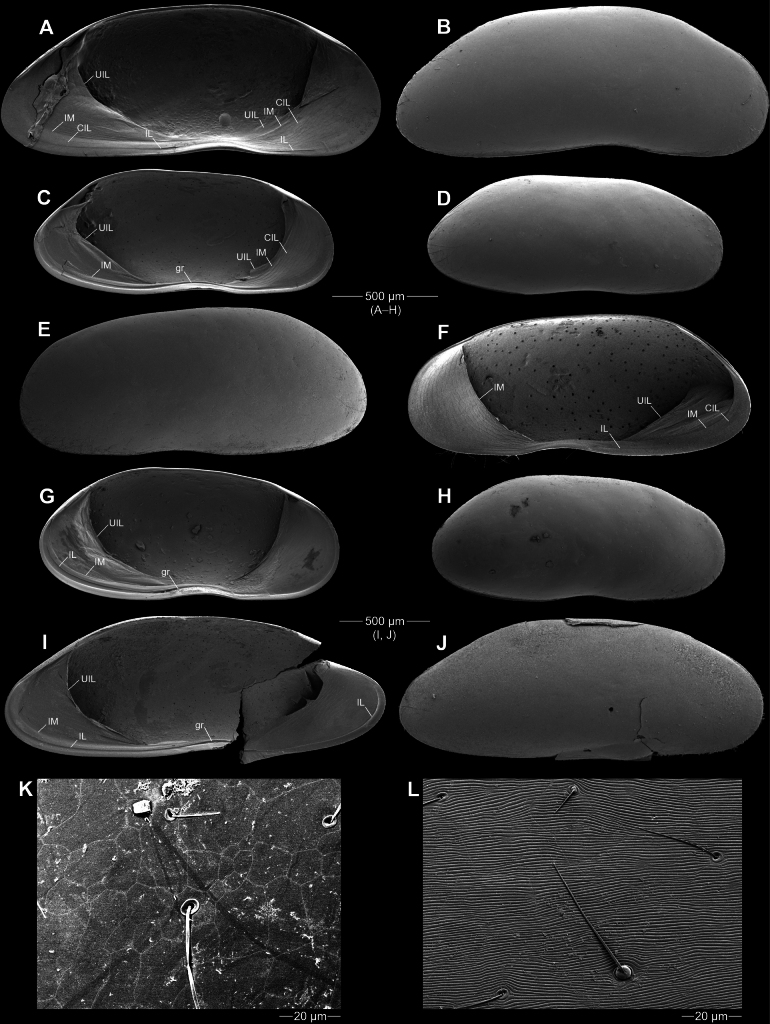
Female valves of five *Stenocypris* species. **A, B**. *S. hirutai* Smith & Kamiya, 2006 (WOC643). **A**. LV, int.; **B**. RV, ext. **C, D**. *S. orientalis* Victor & Fernando, 1981 (WOC493). **C**. LV, int.; **D**. RV, ext. **E, F**. *Stenocypris* sp. 1. (WOC176). **E**. LV, ext.; **F**. RV, int. **G, H**. *Stenocypris* sp. 2. (WOC547). **G**. LV, int.; **H**. RV, ext. **I, J**. *Stenocypris* sp. 3. (WOC485). **I**. LV, int.; **J**. RV, ext.; **K**. Surface detail of *S. orientalis* (WOC122), showing the polygonal pattern; **L**. Surface detail of (**J**), showing the fine striations superimposed with a faint polygonal pattern.

Meisch et al. (2024) listed 40 extant species of *Stenocypris*. Most of these species have long swimming setae that extend at least to the bases of the terminal claws, which offers easy distinction from our new species. The following five species are known to have short swimming setae on the A2 that extend to approx. the midway of the penultimate segment or are shorter: *Stenocypris hirutai*, *Stenocypris ilyophila* Klie, 1932, *Stenocypris intermedia* Klie, 1932, *Stenocypris trapezoides* Klie, 1932, and *Stenocypris tsukagoshii* Smith & Kamiya, 2006. *Stenocypris hirutai* can be distinguished from the new species by its straight, inclined postero-dorsal margin, the absence of a ventral groove on the CIL of the LV (only the inner list is present), and the wide posterior CIL (fig. 2 of Smith and Kamiya 2006 and Figs 3B, 7A, 7Bherein). The posterior part of the LV of *S. ilyophila* (fig. 56 of Klie 1932) is lower than that of the new species (Fig. 2C, F) in lateral view. In dorsal/ventral view, the greatest width of its carapace is situated in the middle (Klie 1932: fig. 57), while being in the posterior area in the new species (Fig. 2E, G–I; Suppl. material 6: fig. 2B). *Stenocypris intermedia* was not illustrated in the original description (Klie 1932), but Petkovski and Meisch (1996) synonymized *S. malcolmsoni lata* Ghetti, 1972 with *S. intermedia*. Based on Ghetti’s (1972) illustrations, this species is very similar to *Stenocypris menghaiensis* sp. nov. in valve outline, and it has a similar narrow posterior CIL (Ghetti 1972: tav. 6d). The greatest width of the carapace of *S. malcolmsoni lata* is situated near the middle (Ghetti 1972: tav. 6a, g). The asymmetrical septa in the antero-ventral part of the two valves (Ghetti 1972: tav. 6a) are not seen in our new species (Suppl. material 6: fig. 2B). Additionally, the cavities between the septa of *S. malcolmsoni lata* do not branch into radial pore canals (Ghetti 1972: tav. 6i) as in the new species (Fig. 4A), although this could be an artifact of the drawing by Ghetti (1972). The limb chaetotaxy of *S. malcolmsoni lata* was incompletely illustrated in Ghetti (1972), which obstructs a more comprehensive comparison with the present species. *Stenocypris trapezoides* has a sub-trapezoidal outline (Klie 1932: fig. 61), which easily distinguishes it from the new species. *Stenocypris tsukagoshii* has a more elongate valve outline (Smith and Kamiya 2006: fig. 2j–l) and the carapace is less inflated in dorsal view (Smith and Kamiya 2006: fig. 2m). Its posterior CIL extends far beyond the septa (Smith and Kamiya 2006: fig. 8a, b).

The lengths of swimming setae of the following species are not known: *Stenocypris curvirami* Lowndes, 1932, *Stenocypris exsiccata* (Vávra, 1897), and *Stenocypris fontinalis* (Vávra, 1895). Nonetheless, they can be distinguished from the present new species by their size and other morphological details: both *S. curvirami* and *S. exsiccata* are > 2 mm in length, much larger than the new species (< 1.5 mm, Table 2). In addition, the valve surface of *S. curvirami* is densely striated (Lowndes 1932: pl. I fig. 2), which differs from the polygonal pattern in the new species (Fig. 2J). The dorsal valve margin of *S. exsiccata* is much more arched, and the posterior margin is much steeper (Vávra 1897: fig. 5.1) compared with the new species (Fig. 2). *Stenocypris fontinalis* is also larger than the new species (1.7 mm) and is more elongate both in lateral and dorsal views (Vávra 1895: fig. 5).

##### Etymology.

Named after Menghai County, where the holotype was collected.

#### Stenocypris hirutai

Taxon classificationAnimaliaPodocopidaCyprididae

Smith & Kamiya, 2006

87D5EBC8-A219-54C7-A8DF-95397B2AAAD2

Figs 3B, 4B1–B2, 7A, 7B, Suppl. material 5: fig. 1B

Stenocypris hirutai Smith & Kamiya, 2006: 337–339, figs 2f–i, 6, 7.

##### Localities.

Three ditches (Y240, Y241, Y507), two rivers (Y258, Y509), five streams (Y262, Y511, Y512, Y529, Y531), and a pond (Y329) (Fig. 1, Table 1).

##### Material examined.

Twenty dissected females (Table 2).

##### Dimensions

**(based on dissected females)**. LV L 1491–1954 μm (*n* = 19), H/L 0.391–0.420 (*n* = 18). RV (*n* = 19) L 1458–1923 μm, H/L 0.389–0.412. LV ~ 30 μm longer than RV of same individual (Table 2).

##### Brief description.

Small- to medium-sized *Stenocypris* with arched dorsal margin and wide posterior CIL (Figs 3B, 7A). In interior view, LV with inner list accompanied by weakly expressed ventral groove along CIL (Fig. 7A). In transmitted light, each septal cavity branching into up to ca six long radial pore canals (Fig. 4B1–B2). Valve surface with polygonal pattern under high magnification of SEM (not illustrated but similar to that of *S. orientalis* as shown in Fig. 7K). A2 swimming setae short, extending to midway of penultimate segment or shorter (Table 3). For a more detailed description, see Smith and Kamiya (2006).

**Table 3.** Summary of the morphological differences of the *Stenocypris* species investigated in this study.

**Characters**	***Stenocypris menghaiensis* sp. nov**.	***S. hirutai* Smith & Kamiya, 2006**	***S. orientalis* Victor & Fernando, 1981**	***Stenocypris* sp. 1**	***Stenocypris* sp. 2**	***Stenocypris* sp. 3**
Width of posterior CIL	Narrow, only slightly beyond septa	Wide, far beyond septa	Narrow, only slightly beyond septa	Intermediate	Narrow, only slightly beyond septa	Narrow, only slightly beyond septa
Ventral groove on CIL of LV	Well-developed groove, widening anteriorly and posteriorly	Short indistinct groove	Well-developed groove, widening anteriorly and posteriorly	na	Well-developed groove, widening anteriorly and posteriorly	Well-developed groove, becoming step anteriorly
Striations on valve surface	Absent	Absent	Absent	Absent	Absent	Present
Length range of LV (μm)	1290–1420	1490–1950*	1390–1760	~ 1660	~ 1430	~ 2060
H/L ratio of LV	0.44–0.45	0.39–0.42	0.41–0.44	~ 0.43	~ 0.44	~ 0.39
Extension of swimming setae on A2	To ~ midway of penultimate segment	To ~ midway of penultimate segment or even shorter	To ~ tips of claws	To ~ midway of penultimate segment	To ~ bases of claws	Slightly beyond tips of claws
Length ratio of h3/h1 setae of L6	2.15–2.58	1.48–2.43	1.66–3.07	~ 1.79	~ 2.22	~ 1.63
Length ratio between A1 3^rd^ segment seta and LV	0.013–0.017	0.011–0.020	0.011–0.015	~ 0.011	~ 0.012	~ 0.013

*The Japanese specimens of this species described by Smith and Kamiya (2006) were smaller, being ~ 1300 μm.

##### Remarks.

The specimens from Yunnan are notable for their large size range (length 1490–1950 μm), with the largest specimens somewhat longer than previous reports (e.g., Smith and Kamiya 2006: length 1250–1430 μm, Ma and Yu 2018: length 1440–1670 μm). The Yunnan specimens also differ a little in the lateral outline of the carapace.

*Stenocypris hirutai* is widespread in East Asia, with records from Japan, Korea, and China (Smith and Kamiya 2006; Smith 2011; Smith et al. 2014; Ma and Yu 2018; Smith and Chang 2022), usually associated with lotic habitats (but see Discussion below).

#### Stenocypris orientalis

Taxon classificationAnimaliaPodocopidaCyprididae

Victor & Fernando, 1981

5933C2E1-EAD9-5D91-ACDB-8FCA83FECB41

Figs 3C, 4C1–C2, 7C, 7D, 7K, Suppl. material 5: fig. 1C

Stenocypris orientalis Victor & Fernando, 1981: 158–160, figs 46–60.
Stenocypris major (Baird, 1859) – Zhai et al. 2023: 5323, Table 1, Table 2 (part), fig. 16D.
Stenocypris major (Baird 1859) – Zhai et al. 2024: 4, 6, figs 2F, 3, 4C, 5C, D.

##### Localities.

Three rice fields (Y134, Y136, Y250), a wet barren field (Y159), and a mountain stream feeding the Nu River (Y255) (Fig. 1, Table 1).

##### Material examined.

Nine dissected females (Table 2).

##### Dimensions

**(based on dissected females)**. LV (*n* = 7) L 1389–1763 μm, H/L 0.409–0.440. RV (*n* = 6) L 1358–1475 μm, H/L 0.405–0.432. LV ~ 30 μm longer than RV of same individual (Table 2).

##### Brief description.

Medium-sized *Stenocypris* with nearly straight dorsal margin and narrow posterior CIL (Victor and Fernando 1981; Figs 3C, 7C, 7D). In interior view, LV with well-developed ventral groove along CIL (Fig. 7C). In transmitted light, each septal cavity branching into short radial pore canals (Fig. 4C1–C2). Valve surface with polygonal pattern under high magnification of SEM (Fig. 7K). A2 swimming setae long, extending to approx. tips of terminal claws (Table 3). For a more detailed description, see Victor and Fernando (1981) and Table 3.

##### Remarks.

*Stenocypris orientalis*, *Stenocypris viridis* Okubo, 1990, *Stenocypris bolieki* Ferguson, 1962, and *Stenocypris hislopi* Ferguson, 1969 are four imperfectly known species that closely resemble one another. Differences are subtle and need confirmation with redescriptions and analyses of intraspecific variation. We tentatively assign our specimens to *S. orientalis* due to the overall similarities to the original description, especially the lateral outlines of the valves, and the micro-ornamentation (polygons) on the carapace.

Zhai et al. (2023, 2024) identified *S. major* from Yunnan; however, after re-examination of the specimens, we identify them as *S. orientalis*. These specimens differ from *S. major* (see Moonchaisook and Savatenalinton 2020) by the less elongate valve outline and the lack of fine striations on the valve surface (Figs 3C, 7C, 7D, 7K).

*Stenocypris orientalis* has been reported from the Philippines, West Malaysia, Thailand, and China (Victor and Fernando 1981; Savatenalinton 2014; Ma and Yu 2018, 2020; Zhai et al. 2023, 2024).

#### Stenocypris

Taxon classificationAnimaliaPodocopidaCyprididae

sp. 1

033157C7-BCCF-519F-B5A6-806DFCFC8AEF

Figs 3D, 4D1, D2, 7E, 7F, Suppl. material 5: fig. 1D

##### Locality.

Site Y255 (Fig. 1, Table 1), a mountain stream feeding the Nu River.

##### Material examined.

One dissected female (WOC176) (Table 2).

##### Dimensions.

LVL 1662 μm, H/L 0.425. RVL 1640 μm, H/L 0.413. LV ~ 20 μm longer than RV (Table 2).

##### Brief description.

Carapace medium to large. Dorsal margin nearly straight, slightly sloping down from maximum height located posterior of mid-length. Anterior margin broadly, but slightly unevenly, rounded. Ventral margin concave. Posterior margin more tightly curved than anterior margin, with apex below mid-height, upper section slightly curved. CIL wide anteriorly, narrower but relatively wide posteriorly, with list on ventral part of RV. Natatory setae of A2 reaching to approx. midway of penultimate segment (Table 3).

##### Remarks.

This species is similar in lateral view to *S. orientalis*, but is larger and has a wider posterior CIL. Unlike *S. orientalis*, this species has notably shorter A2 natatory setae. As only one specimen was recovered, it is left in open nomenclature.

#### Stenocypris

Taxon classificationAnimaliaPodocopidaCyprididae

sp. 2

D719006D-4EE2-57A7-97CE-2A6F1EEBF75E

Figs 3E, 4E1–E2, 7G, 7H, Suppl. material 5: fig. 1E

##### Locality.

Site Y494 (Fig. 1, Table 1), littoral zone of the Red River.

##### Material examined.

One dissected female (WOC547) (Table 2).

##### Dimensions.

LV L 1416 μm, H/L 0.443. RVL 1385 μm, H/L 0.430. LV ~ 30 μm longer than RV (Table 2).

##### Brief description.

Carapace small. Dorsal margin nearly straight, sloping. Anterior margin broadly rounded. Posterior margin more tightly rounded with apex slightly below mid-height. Postero-dorsal part gently curving to maximum height located posterior of mid-length. Ventral margin with concavity. CIL wide anteriorly, narrow posteriorly. LV with well-developed groove widening anteriorly and posteriorly, grading posteriorly into inner list, running up to apex of posterior margin. A2 natatory setae extending to approx. base of claws (Table 3).

##### Remarks.

This species resembles several others in lateral view, such as *S. hislopi*, and *S. orientalis*, but the overall shape is more rounded, especially the posterior margin. In contrast to similar species, this species has reduced A2 natatory setae. As only one specimen was recovered, it is placed in open nomenclature.

#### Stenocypris

Taxon classificationAnimaliaPodocopidaCyprididae

sp. 3

42E8FDD5-055B-5C57-BC4C-8753C4C42720

Figs 3F, 4F1, F2, 7I, 7J, 7L, Suppl. material 5: fig. 1F1, F2

##### Locality.

Site Y250 (Fig. 1, Table 1), desiccated rice field.

##### Material examined.

One dissected female (WOC485) (Table 2).

##### Dimensions.

LVL 2055 μm, H/L 0.385. RVL 2018 μm, H/L 0.383. LV ~ 40 μm longer than RV (Table 2).

##### Brief description.

Carapace large. Dorsal margin slightly curved, sloping down from maximum height located posterior of mid-length. Anterior margin narrow and relatively tightly curved. Posterior margin tightly curved, more so than anterior margin, apex below mid-height. Ventral margin slightly concave. Outer surface of valves covered with faint polygons (~ 20–40 μm across) and tightly packed striations (~ 1 μm apart and continuing across borders of polygons). CIL wide anteriorly, narrow posteriorly. Left valve with well-developed groove running along ventral part of CIL, transforming into inner lists at each end, reaching halfway up posterior margin and near top of anterior margin. Natatory setae of A2 reaching slightly beyond tips of claws (Table 3).

##### Remarks.

This species is similar to *S. major* with its large size, an inner list accompanied by a distinct groove on the LV running along the anterior CIL almost up to the hinge area, and fine striations on the carapace (see Triebel 1953; Moonchaisook and Savatenalinton 2020). However, both the anterior and posterior margins are more tightly curved, and the apex of the posterior margin is lower compared to *S. major*, suggesting that it is not conspecific. As only one specimen was recovered, it is left in open nomenclature.

### Principal coordinate analysis

**Figure 8.**
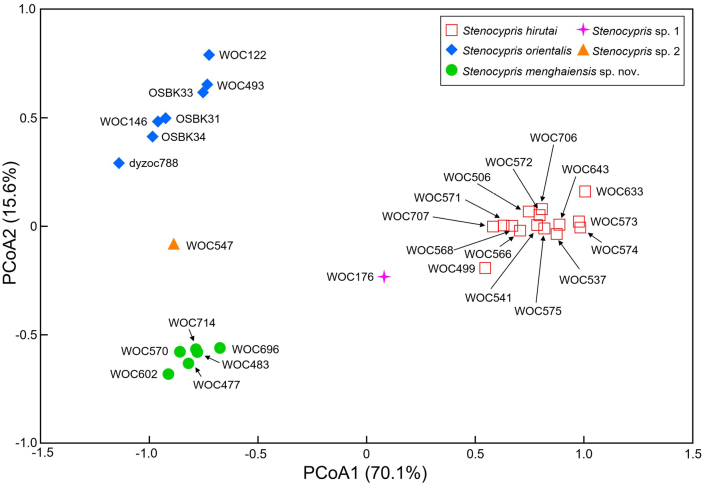
Biplot of Principal Coordinate Analysis (PCoA) of the *Stenocypris* specimens based on their valve and soft-part morphological data (Suppl. material 4). The specimen of *Stenocypris* sp. 3 (WOC485) is not included in the analysis because of the lack of valve outline data (valves broken).

Principal Coordinate Analysis (PCoA) visualizes the intra- and inter-species morphological variabilities of *Stenocypris* (Fig. 8). The first two axes of PCoA capture 70.1% and 15.6% of the variance in the morphological data, respectively, totalling 85.7%. The specimens belonging to five different *Stenocypris* species (the specimen of *Stenocypris* sp. 3 is not included in Fig. 8 because its valves were damaged and the valve outline data are not available) are clearly separated in the biplot: *S. orientalis*, *Stenocypris* sp. 2, and *Stenocypris menghaiensis* sp. nov. are separated from *S. hirutai* and *Stenocypris* sp. 1 by PCoA1, while PCoA2 clearly separates *S. orientalis* and *Stenocypris* sp. 2 from the new species. *Stenocypris hirutai* and *S. orientalis* show considerable intra-species variability, both being rather scattered in the biplot. In particular, the specimens of *S. orientalis* are more scattered despite the small sample size (seven specimens). On the contrary, *Stenocypris menghaiensis* sp. nov. shows stronger morphological consistency, which may be attributed to the fact that the five sampling sites of this species are geographically more restricted compared with the other two species (Fig. 1).

## Discussion

### Habitat

Of the 40 previously described subjective species in the genus *Stenocypris*, only five have reduced natatory setae on the A2 (i.e., not reaching to the distal ends of the largest claws) (see Differential diagnosis of *Stenocypris menghaiensis* sp. nov. above). Reduced natatory setae are a homoeomorphic character seen in various taxa in several lineages, and are associated with relatively low-energy lotic habitats, such as the discharges of springs and riverine habitats; the diminished swimming ability means that individuals remain on the sediment surface, where they are less likely to be swept downstream by currents (Smith 2025). This study includes three additional species with reduced natatory setae, *Stenocypris menghaiensis* sp. nov., *Stenocypris* sp. 1, and *Stenocypris* sp. 2., plus additional reports of *S. hirutai* (which also has reduced natatory setae). The relatively high number of species with reduced natatory setae in this study is probably related to the habitats sampled: rivers, streams, ditches, and a wet field influenced by a stream (Table 1). *Stenocypris hirutai* and *Stenocypris menghaiensis* sp. nov. have mainly been found in flowing waters (Smith and Kamiya 2006; Ma and Yu 2020; Table 1 of the present study), although *S. hirutai* was also found in a pond (site Y329, Table 1). However, *S. hirutai* was frequently found in lentic waters, such as ponds and reservoirs, in the Tiantong area of eastern China (Ma and Yu 2018). These observations suggest that *S. hirutai* adapts to both lotic and lentic habitats. Based on albeit limited data available, it seems that Yunnan Province has four *Stenocypris* lineages that have colonized lotic environments, which perhaps suggests a high colonization rate compared with other regions.

In contrast to *S. hirutai*, *S. orientalis*, which has long natatory setae, has been found mainly in lentic waters, e.g., rice fields, ponds, and puddles (Victor and Fernando 1981; Ma and Yu 2020; this study). However, the occurrence of *S. orientalis* in a stream in our study (Tables 1, 2; only one specimen), as well as a few brooks and a river in Thailand (Savatenalinton 2014: tables 1, 2), indicate that this species could also tolerate lotic environments.

### Biogeography

Previously, seven species of *Stenocypris* were known from China, namely *Stenocypris derupta* Vávra, 1906, *S. hirutai*, *S. major*, *Stenocypris malayica* Victor & Fernando, 1981, *S. orientalis*, *S. viridis*, and *Stenocypris* sp. of Yu (2014) (Chen 1982; Yu et al. 2009; Yu 2014; Ma and Yu 2018, 2020). Thus, the six *Stenocypris* species recovered in this study represent a comparatively large number, and increase the number known in China to 11. Yunnan Province is generally considered to be in the Oriental zoogeographical region (e.g., Zhang 2011; Meisch et al. 2024), although Zhai et al. (2023) noted that the ostracod fauna contains more Palaearctic than Oriental species. The records of *S. hirutai* herein expand the known geographical range of this species into southwest China by ca 800 km from the nearest previous record, on Hainan Island (Ma and Yu 2020). Hainan Island is in the Oriental region, but other previous records of *S. hirutai* are from the Palaearctic region (Japan, Korea, and China), and thus this species may straddle the Palaearctic–Oriental boundary. On the other hand, *Stenocypris orientalis* has previously been found in Yunnan (Zhai et al. 2024, as *S. major*), as well as Hainan Island, the Philippines, West Malaysia, and Thailand (Victor and Fernando 1981; Savatenalinton 2014; Ma and Yu 2020), but not in the Palaearctic. Thus, this species could be an Oriental element of the Chinese ostracod fauna.

### Taxonomy

Since the genus *Stenocypris* was established in 1889 (Sars 1889), ~ 79 elongate, laterally compressed extant cyprids with asymmetrical uropods have been described as *Stenocypris*. Not all species have carapaces with septa, and so many have subsequently been moved to different genera, notably *Chrissia* and *Humphcypris* (e.g., Martens 1997, 2001). Currently, 41 named living species are assigned to the genus, but still some species may not belong there. For example, in the original descriptions of both *Stenocypris caesia* Klie, 1935 and *Stenocypris damasi* Kiss, 1959, septa were not mentioned or figured. Of the remaining species, discrimination is difficult because of a lack of data, particularly of the valves, such as the presence of grooves, inner lists and surface micro-ornamentation, and the scarcity of sexual species (only seven species have males). Fine carapace features are not clearly visible without the assistance of SEM (but see Suppl. material 5), and many species were described before this technology was invented or became widespread. Ironically, the septa, one of the defining characters of the genus, are not visible with SEM, as they are located between the inner and outer lamellae. But the septa are visible with transmitted light microscopy (Fig. 3, Suppl. material 5), and can be taxonomically useful. In some species, the septa are noticeably longer in the antero-dorsal region compared with the rest of the anterior margin (e.g., *S. orientalis*, *Stenocypris menghaiensis* sp. nov.) (Fig. 3), while for others, the septa are more or less equal in length, or show a gradual change, around the anterior margin [e.g., *S. major* (see Triebel 1953), and *Stenocypris sketi* Petkovski & Meisch, 1996]. Another potentially helpful feature is the length of the radial pore canals that originate from the space between the septa. Some species, for example *S. major* (Triebel 1953), *S. orientalis* (Fig. 4C1–C2), and *Stenocypris* sp. 3 (Fig. 4F1), have relatively short radial pore canals, while these structures are noticeably long in others such as *Stenocypris menghaiensis* sp. nov. and *S. hirutai* (Fig. 4A1–B2). However, both the septa and the radial pore canals continue to develop after the final molt as specimens mature, so adults of the same species can show marked variabilities in these marginal structures (Wouters 1999; Smith et al. 2011; Moonchaisook and Savatenalinton 2020). As a result, more specimens should be examined for these characters in the future.

The appendages generally appear to be conservative within the genus, although for most species, the appendages are not fully described. The length of natatory setae on the antennae, and the abnormally long h3 seta in a couple of species (*S. malayica* and *Stenocypris sancari* Külköylüoğlu et al., 2021) are examples of appendage characters that do show some variation (Victor and Fernando 1981; Külköylüoğlu et al. 2021). All species in this study have h3 setae of ‘normal’ lengths, but there is some variation in the length ratios of h3 and h1, both within and between species (Table 3). Whether these ratios have any taxonomic significance requires further research. Overall, the taxonomy of the genus can only be resolved with redescriptions (of type specimens when possible), focusing on the fine details of the valves, such as grooves, lists, widths of CILs, micro-ornamentation and valve outlines, and subtle details of the appendages. Moreover, our PCoA analysis (Fig. 8) shows the potential of quantified morphological parameters in species distinction in lineages such as *Stenocypris*, where males are rare, and females of different species have identical chaetotaxy. In such a case, in addition to the few types of morphological hiatus (such as the extension of the swimming setae and the widths of the posterior CIL), the length variations of the setae and the segments may also be utilized. By measuring a number of specimens from each species, the intra-specimen variability can be evaluated, establishing specimen cluster patterns for different species, which can lend support to species distinctions. However, such a method is time- and labor-consuming (cf. Suppl. materials 1–4). In order to facilitate its application, morphological parameters should be properly selected to effectively capture the morphological variation. Therefore, selecting good parameters should be a topic in future studies. Moreover, future investigations should include more specimens from different types of habitats and different regions to capture more intra-specific variation, so that the morpho-space of each species can be more accurately visualized in analyses such as PCoA.

## Supplementary Material

XML Treatment for Stenocypris menghaiensis

XML Treatment for Stenocypris hirutai

XML Treatment for Stenocypris orientalis

XML Treatment for Stenocypris

XML Treatment for Stenocypris

XML Treatment for Stenocypris

## Additional information

### Artificial Intelligence (AI) use

The authors accept full responsibility for the content of the manuscript, including the disclosure of any use of AI.

No AI tools were used in the preparation of this manuscript.

### Author ORCIDs

D. Zhai https://orcid.org/0000-0001-6312-851X

R.J. Smith https://orcid.org/0000-0002-9888-5323

H. Mai https://orcid.org/0000-0002-3157-263X

P. Jiang https://orcid.org/0009-0007-9193-3883

K. Zeng https://orcid.org/0009-0003-5111-2205

### Data availability

All of the data that support the findings of this study are available in the main text or Supplementary Information.

## Supplementary materials

Supplementary material 1 Stenocypris soft part measurements Data type xlsx https://binary.pensoft.net/file/1712692This dataset is made available under the Open Database License (http://opendatacommons.org/licenses/odbl/1.0/). The Open Database License (ODbL) is a license agreement intended to allow users to freely share, modify, and use this Dataset while maintaining this same freedom for others, provided that the original source and author(s) are credited. Dayou Zhai, Robin James Smith, Huijuan Mai, Ping Jiang, Kewei Zeng

Supplementary material 2 LV outline data Data type xlsx https://binary.pensoft.net/file/1712693This dataset is made available under the Open Database License (http://opendatacommons.org/licenses/odbl/1.0/). The Open Database License (ODbL) is a license agreement intended to allow users to freely share, modify, and use this Dataset while maintaining this same freedom for others, provided that the original source and author(s) are credited. Dayou Zhai, Robin James Smith, Huijuan Mai, Ping Jiang, Kewei Zeng

Supplementary material 3 Dissimilarity matrices based on valve outlines and soft-part measurements, and quantified data of morphologies Data type csv https://binary.pensoft.net/file/1712694This dataset is made available under the Open Database License (http://opendatacommons.org/licenses/odbl/1.0/). The Open Database License (ODbL) is a license agreement intended to allow users to freely share, modify, and use this Dataset while maintaining this same freedom for others, provided that the original source and author(s) are credited. Dayou Zhai, Robin James Smith, Huijuan Mai, Ping Jiang, Kewei Zeng

Supplementary material 4 Forwarded normalized data for PCoA, selected from Suppl. material 3 Data type csv https://binary.pensoft.net/file/1712695This dataset is made available under the Open Database License (http://opendatacommons.org/licenses/odbl/1.0/). The Open Database License (ODbL) is a license agreement intended to allow users to freely share, modify, and use this Dataset while maintaining this same freedom for others, provided that the original source and author(s) are credited. Dayou Zhai, Robin James Smith, Huijuan Mai, Ping Jiang, Kewei Zeng

Supplementary material 5 Oblique-dorsal views of the female LVs of *Stenocypris* species, showing the groove/inner list Data type jpg Explanation note The specimens in A–F correspond to those in Fig. 3A–F, respectively. (A) *Stenocypris menghaiensis* sp. nov. (WOC483, holotype); (B) *S. hirutai* Smith & Kamiya, 2006 (WOC541); (C) *S. orientalis* Victor & Fernando, 1981 (OSBK31); (D) *Stenocypris* sp. 1 (WOC176); (E) *Stenocypris* sp. 2 (WOC547); (F1–F2) *Stenocypris* sp. 3 (WOC485), fragments of posterior (F1) and anterior (F2) parts. Scale applies to all panels. https://binary.pensoft.net/file/1712696This dataset is made available under the Open Database License (http://opendatacommons.org/licenses/odbl/1.0/). The Open Database License (ODbL) is a license agreement intended to allow users to freely share, modify, and use this Dataset while maintaining this same freedom for others, provided that the original source and author(s) are credited. Dayou Zhai, Robin James Smith, Huijuan Mai, Ping Jiang, Kewei Zeng

Supplementary material 6 Two undissected specimens of *Stenocypris menghaiensis* sp. nov. Data type jpg Explanation note (A) WOC723, left view (fecal pellet displaced during preparation); (B) WOC722, ventral view, anterior to left (soft parts somewhat deteriorated). For detailed views of the marginal zone, see Fig. 4. https://binary.pensoft.net/file/1712697This dataset is made available under the Open Database License (http://opendatacommons.org/licenses/odbl/1.0/). The Open Database License (ODbL) is a license agreement intended to allow users to freely share, modify, and use this Dataset while maintaining this same freedom for others, provided that the original source and author(s) are credited. Dayou Zhai, Robin James Smith, Huijuan Mai, Ping Jiang, Kewei Zeng
