# Supplementary figures and images for: On Stenocypris (Crustacea, Ostracoda) species from Yunnan Province, southwestern China, with a description of a new species
Source: Zookeys. 2026 Jul 10;1284:271–97. doi: 10.3897/zookeys.1284.195403 (PMC13379715; doi:10.3897/zookeys.1284.195403)

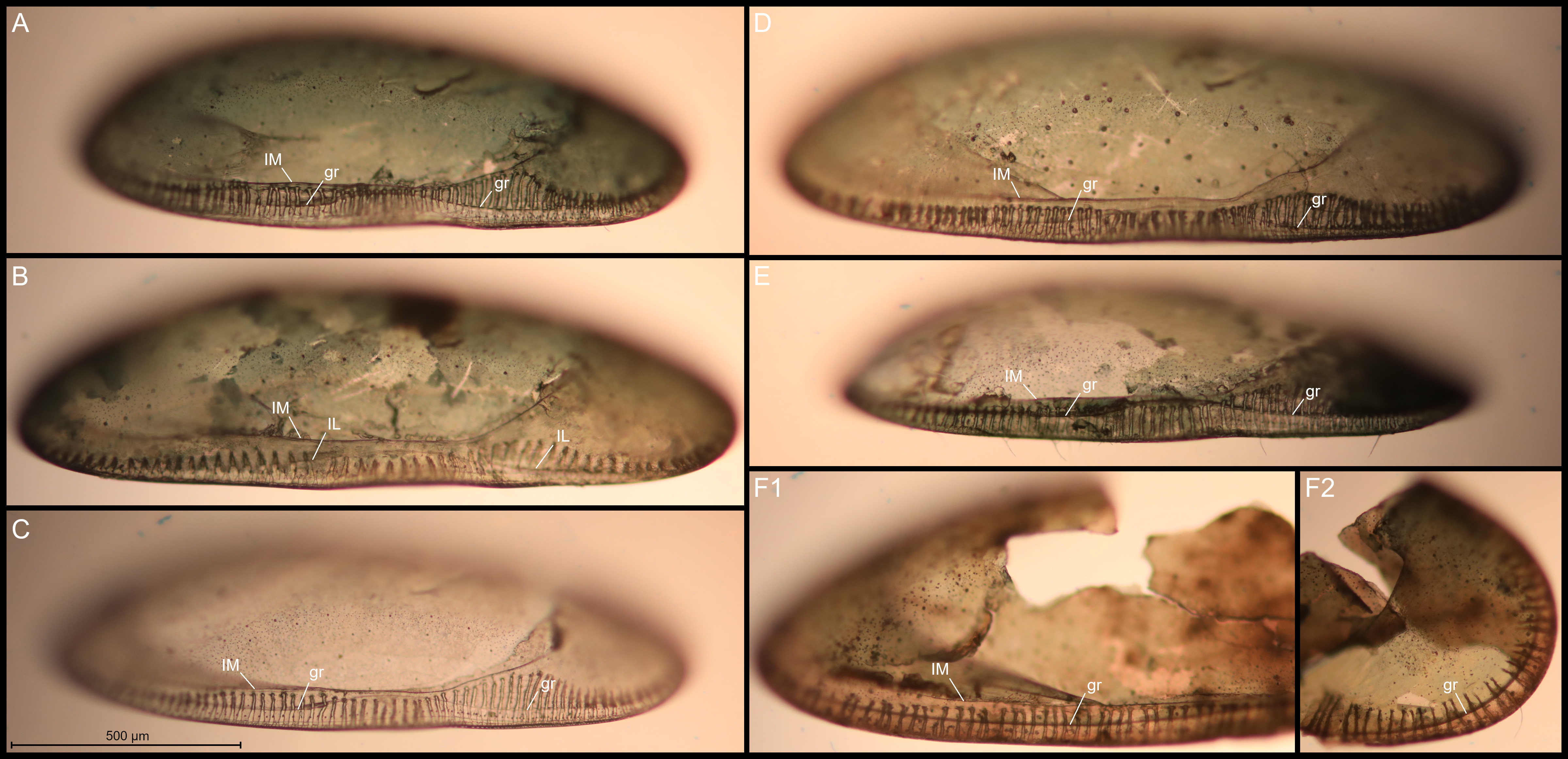

Supplement: Supplementary material 1 — Stenocypris soft part measurements [file zookeys-1284-271_article-195403__-s001.jpg]

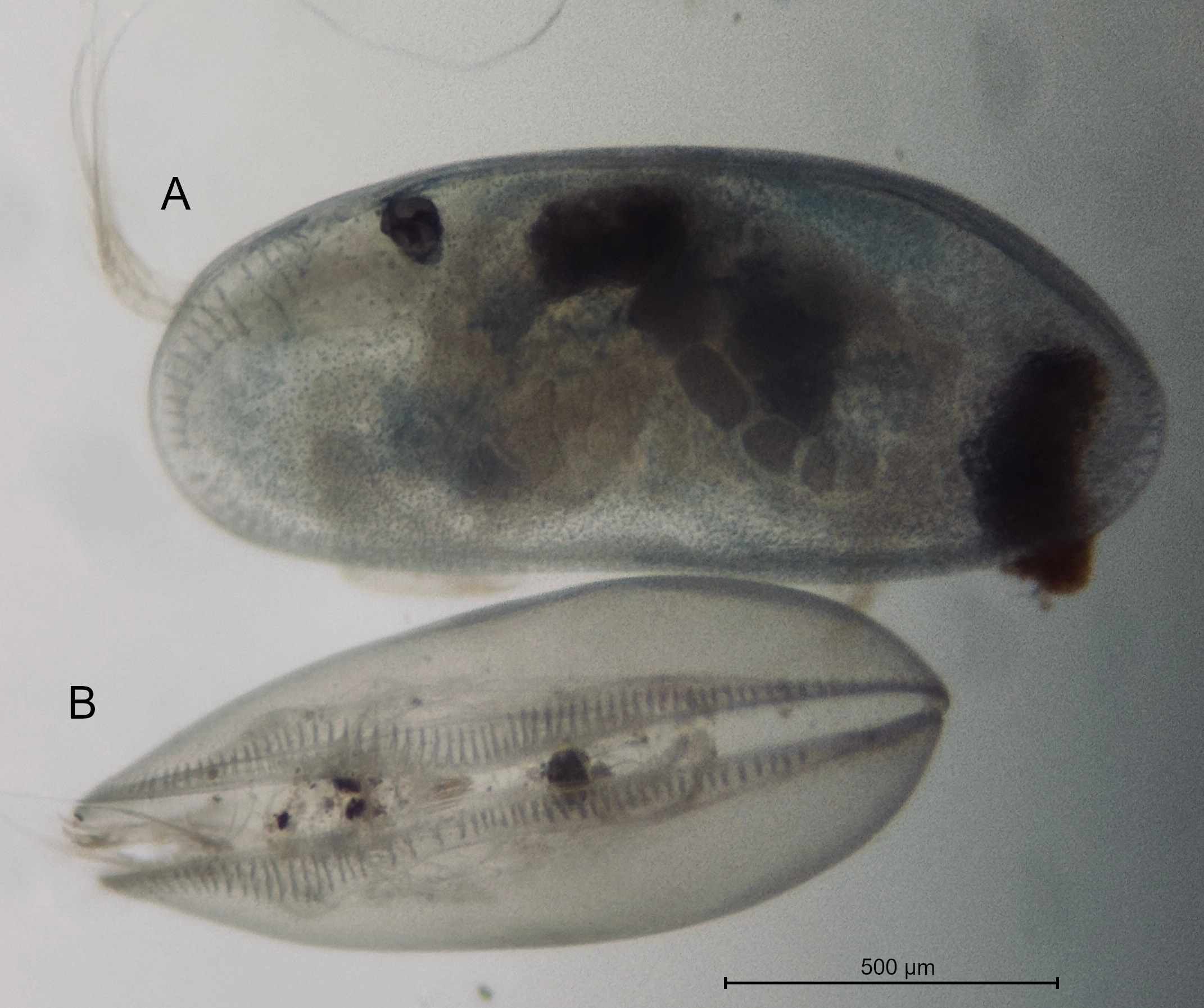

Supplement: Supplementary material 2 — LV outline data [file zookeys-1284-271_article-195403__-s002.jpg]
